# Sex-Specific Dietary Patterns and Social Behaviour in Low-Risk Individuals

**DOI:** 10.3390/nu15081832

**Published:** 2023-04-11

**Authors:** Daniel Engler, Renate B. Schnabel, Felix Alexander Neumann, Birgit-Christiane Zyriax, Nataliya Makarova

**Affiliations:** 1 Department of Cardiology, University Heart & Vascular Center Hamburg-Eppendorf (UHZ), University Medical Center Hamburg-Eppendorf (UKE), 20246 Hamburg, Germany; 2 German Center for Cardiovascular Research (DZHK), Partner Site Hamburg/Kiel/Lübeck, 20246 Hamburg, Germany; 3 Preventive Medicine and Nutrition, Midwifery Science—Health Services Research and Prevention, Institute for Health Services Research in Dermatology and Nursing (IVDP), University Medical Center Hamburg-Eppendorf (UKE), 20246 Hamburg, Germany

**Keywords:** social behaviour and preferences, dietary patterns, lifestyle, atrial fibrillation, cardiovascular disease, sex-specific differences

## Abstract

Dietary and social behaviour are non-medical factors that influence health outcomes. Non-communicable diseases are related to dietary patterns. To date, little is known about how social behaviour is associated with health-related dietary patterns, and, in particular, we lack information about the role of sex within this possible relation. Our cross-sectional study investigated associations between dietary patterns and social behaviour including personality traits (self-control, risk taking), political preferences (conservative, liberal, ecological, social) and altruism (willingness to donate, club membership, time discounting) in men and women. We performed sex-specific correlation analyses to investigate relationships between dietary patterns based on self-reported protocols from the Mediterranean Diet Adherence Screener (MEDAS) and the validated Healthy Eating Index (HEI) from the EPIC Study and a self-reported social behaviour questionnaire. In linear regression models, we analysed associations between dietary and social behaviour patterns. Sex differences were measured by interaction analysis for each social behaviour item. The study sample consisted of N = 102 low-risk individuals. The median age of the study participants was 62.4 (25th/75th percentile 53.6, 69.1) years, and 26.5% were women. Analyses showed that a lower HEI score was correlated with a higher BMI in both women and men. MEDAS and HEI showed a positive correlation with each other in men. In men, a higher MEDAS showed a positive correlation when they estimated their ability as high, with the same for self-control and preference for ecological politics and MEDAS. A weak negative correlation has been shown between men with a preference for conservative politics and MEDAS. HEI showed a positive significant correlation with age in men. Male participants without club membership scored significantly higher in the HEI compared to non-members. A negative correlation was shown for time discounting in men. Linear regression models showed positive associations between preferences for ecological-oriented politics and nutrition for both HEI and MEDAS. No sex interactions were observed. We faced a few limitations, such as a small sample size, particularly for women, and a limited age spectrum in a European cohort. However, assuming that individuals with a preference for ecological-oriented politics act ecologically responsibly, our findings indicate that ecological behaviour in low-risk individuals might determine, at least in part, a healthy diet. Furthermore, we observed dietary patterns such as higher alcohol consumption in men or higher intake of butter, margarine and cream in women that indicate that women and men may have different needs for nutritional improvement. Thus, further investigations are needed to better understand how social behaviour affects nutrition, which could help to improve health. Our findings have the potential to inform researchers and practitioners who investigate the nature of the relationship between social behaviour and dietary patterns to implement strategies to create first-stage changes in health behaviour for individuals with a low cardiovascular risk profile.

## 1. Introduction

### 1.1. Dietary Patterns and Cardiovascular Disease

Cardiovascular disease (CVD) continues to be the leading cause of mortality in developed countries and is one of the primary leading causes worldwide [1]. The burden of CVD is attributable to multiple risk factors for disease development [1]. While understanding the underlying medical causes of death, it is important to investigate lifestyle and social drivers of the disease. Dietary patterns have been shown to have a fundamental role in the prevention of CVD [2]. A meta-analysis showed a 22% CVD risk reduction for individuals scoring high in diet quality assessments [3], 19–28% in women and 14–26% in men. Current indices such as the Mediterranean Diet Adherence Screener (MEDAS) showed associations with dietary patterns and several health benefits, including a reduction in total mortality [4] and a decrease in metabolic syndrome risk [5]. Poor diet is a known risk factor for overweight and obesity and is associated with the development of CVD. It has been proposed that personality may be linked to dietary patterns [1,6]. Findings from different studies show a positive association between openness to experience and the consumption of fruits and vegetables and between healthy eating habits [7,8].

### 1.2. Relation between Social Behaviour, Dietary Pattern and Cardiovascular Health

It has been proposed that personality may be linked to dietary patterns [1,6]. Findings from different studies show a positive association between openness to experience and the consumption of fruits and vegetables and between healthy eating habits [7,8]. Individual social circumstances can influence the type and variety of food consumed in multiple pathways and thereby impact health. Eating behaviour is influenced by social context and, for instance, it is more likely to follow an eating norm if it is perceived to be relevant based on social comparison [9]. Thus, norms of healthy eating are set by the behaviour of other individuals. These social norms have a powerful effect on both food choices and intake [10]. Furthermore, psychosocial mechanisms such as social support or isolation, social influence or independency, social engagement or separation and the access to resources and information are involved in food choices [11]. However, little is known about social behaviour such as personality traits (self-control, risk taking), political preferences (conservative, liberal, ecological, social) and altruism (willingness to donate, club membership, time discounting) in relation to adherence to dietary recommendations. Previous studies have shown that prosocial factors such as conscientiousness are associated with a number of health-promoting behaviours that include a reduced BMI, avoiding alcohol-related harm, binge drinking and smoking and adherence to medication regimens [12,13,14]. Moreover, an individual participant meta-analysis showed that conscientiousness appears to be related to mortality risk across populations [15].

Evidence suggests that risk factors such as conscientiousness, self-control and risk taking influence health and illness by shaping barriers and facilitators to access to care and health-related behaviours [16].

### 1.3. The Role of Sex-Specific Dietary Patterns for Social Behaviour and Health

Women and men differ in their different types of social relationships. Women, older individuals and more educated individuals consider health aspects more important than other factors, whilst men consider the taste of food and eating habits as the main determinants of food choices [17]. In a survey of attitudes to food, nutrition and health, results indicated that factors such as quality/freshness, price, taste, trying to eat healthily and the eating habits of family members have an important influence on food choices [18].

However, social attitudes and beliefs vary in individuals; to date, little is known about how social behaviour is associated with health-related dietary patterns. In particular, we lack information about the role of sex within this possible interrelation. This evidence might be applicable for social behaviour-based prevention strategies, e.g., concerning sex-specific dietary habits, the evidence is largely lacking.

### 1.4. Aim of the Study

Unhealthy dietary patterns are important drivers of an increased cardiovascular risk. They may vary between men and women and social determinates. To date, research examining the relationship of social behaviour with healthy dietary patterns has been limited. Therefore, we investigated individuals with a low risk profile to identify possible relationships between social behaviour factors and sex-specific dietary patterns.

## 2. Materials and Methods

### 2.1. Design, Setting and Participants

#### 2.1.1. AHRI Study

The AFHRI cohort is a prospective, monocentric, clinical cohort study, to improve the prediction of personal risk for AF. In the current study, a sub-sample of atrial fibrillation (AF) patients with a low cardiovascular risk factor burden—in particular, no prevalent cardiovascular disease, thyroid dysfunction or cancer—was invited for participation (AFHRI-C). The AFHRI-C was planned as a case–control study. Case participants of the study needed to have AF without other cardiovascular diseases. The population-based controls from the Hamburg City Health Study (HCHS) had to fulfil the following criteria: no cardiovascular disease and limited or no risk factors that are related to AF and no known AF. All participants personally signed informed consent forms.

#### 2.1.2. HCHS Study

The Hamburg City Health Study (HCHS) is a single-centre, prospective, epidemiologic cohort study with an emphasis on imaging to improve the identification of individuals at risk for major chronic diseases, to improve early diagnosis and survival. The enrolled participants were selected from a statistical sample provided by the local residents’ registration office. Participants between 45 and 74 years of age from the general population of Hamburg, Germany are included in the study.

For our analysis, we combined the participants from the AFHRI cohort matched with a sub-sample from the population-based Hamburg City Health Study (HCHS). Matching of individuals was based on age, sex and risk factors [19]. Detailed information on both cohorts, their design and the matching approach was published previously [20].

### 2.2. Variables, Measurements and Processes

Questionnaire data and peripheral venous blood were acquired on the day of enrolment. The participants’ characteristics were collected through a questionnaire administered by a healthcare professional, and from patient records.

A three-day dietary record was assessed before their first study participation appointment. A standardised procedure during data collection, with precise questions administered by a study nurse, was applied. The food data were analysed in terms of energy and nutrient intake and adherence to the dietary patterns of the Mediterranean Diet Adherence Screener (MEDAS) [21] and the Healthy Eating Index (HEI) [21,22]. The 14-item MEDAS includes the frequency of food consumption (olive oil, vegetables, fruits, red meat, animal fats, carbonated drinks, red wine, fish/seafood, legumes, nuts, commercial foods and traditional Mediterranean dishes with tomato sauce) as well as the preferred cooking fat and meat consumed. Each item was scored zero or one depending on whether the item-specific criteria were met, resulting in a score between 0 and 14. For the HEI, we used the edition validated in German. The HEI scores five food groups from 0 to 10 (cereal and potatoes; dairy; meat, sausages, fish and eggs; fat or oil; sweets and foods high in fat) and three food groups from 0 to 20 (vegetables; fruits; beverages), allowing total scores between 0 and 110 points. Detailed information of the clinical visits, ECG registration and collection of the nutrition data have been published previously [20]. The following behaviour and preference items were used for analysis: personality traits of risk taking by survey questions “Are you generally a risk-taker, or do you try to avoid risk?”; self-control “When I set my mind to something, I follow through”; religiousness “How strongly religious do you consider yourself to be?”; political preferences (conservative, liberal, ecological, social) “I identify myself with …-oriented politics”; club membership “Are you an active and/or passive member in a club?”; and time discounting. Time discounting means the willingness to give up something at present in order to benefit from something in the future. Each participant received an expense allowance of EUR 50 for study participation. To assess altruistic behaviour, we asked the participants if and how much they were willing to donate to the United Nations Children’s Fund (UNICEF). For the analysis, we used the continuous variable of the willingness to donate scaled from EUR 0 (no funding—less altruistic behaviour) to EUR 50 (maximum funding—more altruistic behaviour). All variables of the present analysis and detailed definitions of the five behaviour preferences are explained in Supplementary Table S1.

### 2.3. Data Handling

Of N = 104, two participants were excluded from the analysis due to missing values of more than 50% in the behaviour questionnaire. The variables were tested using Shapiro–Wilk tests for normal distribution and visualisations such as scatter plots and box plots for outliers. Behaviour preferences and the willingness to donate showed a normal distribution. After checking whether missing values were distributed randomly, using Little’s MCAR Test (chi^2^ (69) = 69.91, *p* = 0.245), we performed a multiple data imputation by single value regression analysis with five iterations for the imputation [23] and aggregated these into a pooled value using the Bar procedure [24]. Missing values were imputed for income (14), resting heart rate (11), NT-proBNP (8) and weight (2). No impact of the imputation on our results was detected. Details of the iteration steps for the imputation were published perilously [20]. We used SPSS Statistics (version 27.0; IBM Corp., Armonk, NY, USA) for all the analyses.

### 2.4. Statistical Analysis

Categorical variables of participant characteristics are presented by their absolute and relative frequencies, and Pearson’s chi-square and Fisher’s exact tests were used to determine subgroup comparisons. Shapiro–Wilk tests indicated that most of the continuous variables significantly varied from a normal distribution. Therefore, continuous variables are reported as the median and the first and third quartiles. The Mann–Whitney U test was applied for group comparisons. Furthermore, bivariate correlations of variables were assessed using Spearman’s rho test. To analyse sex-specific dietary patterns, we applied the standardised test statistics of Pearson’s Chi.

**Figure 1:**
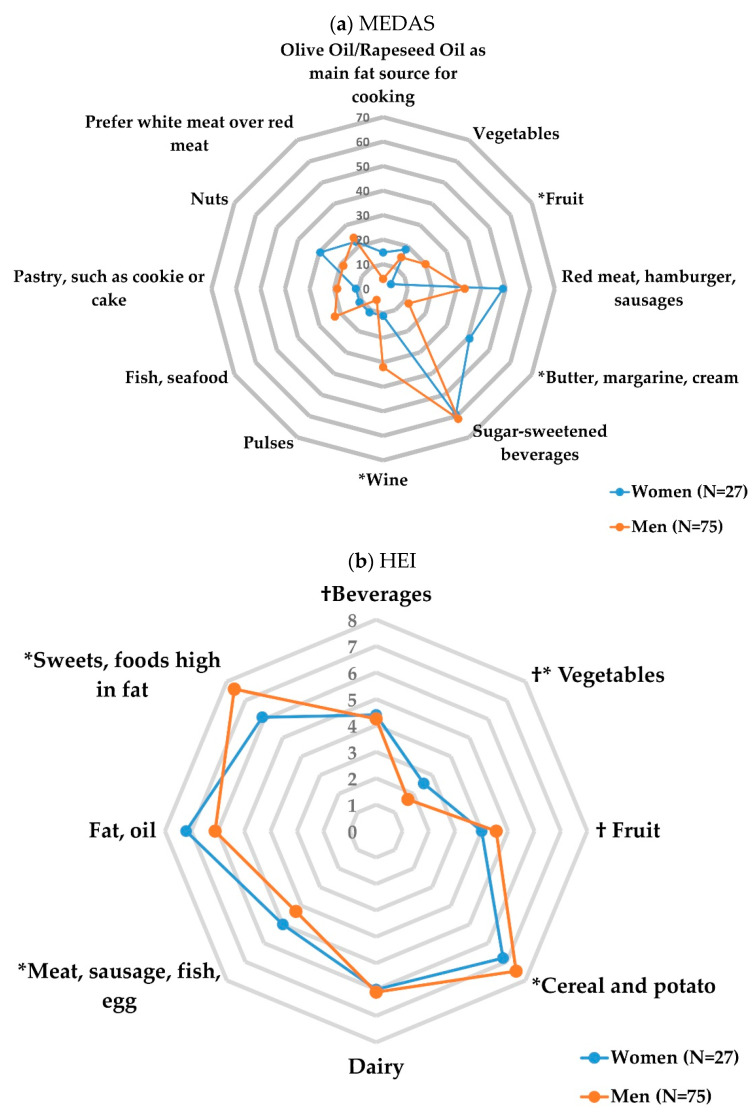
Sex differences in the food items of the Mediterranean Diet Adherence Screener (MEDAS) and the Healthy Eating index (HEI). Dietary patterns of men (N = 75) and women (N = 27) were determined according to average scores achieved for items in the Mediterranean Diet Adherence Screener (MEDAS) and items in the Healthy Eating Index (HEI). For MEDAS, the category “olive oil, rapeseed oil” was excluded as no participant scored it. Scores are presented in percentages for MEDAS and as the average median for items in the Healthy Eating Index (HEI). The scores ranged within 0–10 or 0–20 points depending on the item. † maximum points downscaled from 20 to 10 points. * *p* < 0.05 for significance. Detailed numerical results are presented in Supplementary Table S2 for MEDAS and Supplementary Table S3 for HEI.

To analyse the association between behaviour preferences and the indices (MEDAS and HEI), we performed multiple linear regression analysis. We analysed outliers by cook test and tested variables for autocorrelation by Durban–Watson. Multi-collinearity was tested by VIF. Additionally, we performed interaction analysis to investigate a possible difference or similaritiy with sex. All possible confounders were tested for significance within the model. The variable of club membership was significantly correlated with social politics; thus, we excluded membership from the regression analysis. Results of the regressions are presented as beta and their 95% lower and upper confidence intervals (CI). R^2^ was used to describe the regression model’s fit. Linear regression models with beta coefficients and interaction analyses were performed to show the association between social behaviour and both HEI and MEDAS. The significance level was set to *a* < 0.05. All statistical tests were computed using SPSS Statistics (version 27.0; IBM Corp., Armonk, NY, USA) and Microsoft Excel 2013 for the visualisation in Figure 1 (Microsoft, Redmond, WA, USA).

## 3. Results

### 3.1. Characteristics of Study Participants

Overall, N = 102 participants with a median age of 62.4 (26.5% women) were included in the analysis. Significant sex differences were shown for income (z = 10.3, *p* = 0.008). Men had a significantly higher income compared to women. Dyslipidaemia was more frequent in women (z = 6.1, *p* = 0.014). Women reported significantly less alcohol intake (z = 4.3, *p* = 0.069). The social behaviour assessment showed significant results for active club membership in men (z = 10.7, *p* = 0.004) and a higher preference for conservative politics (z = 17.8, *p* = 0.032) than in women. More detailed information on study participants is provided in Table 1.

### 3.2. Sex-Specific Dietary Patterns

For MEDAS, women scored significantly lower for the food groups “fruit” (z = 4.0, *p* = 0.046) and “wine” (z = 4.6, *p* = 0.035) compared to men. The intake of butter, margarine and cream was higher in women compared to men (z = 10.3, *p* = 0.001). For the HEI, only the food intake of vegetables was significantly higher when reported by women compared with the male participants (z = 2.8, *p* = 0.005). The overall HEI score did not show differences between the groups Figure 1.

### 3.3. Correlation Analysis

For MEDAS, no significant correlations were shown in women. The HEI showed a significant positive correlation for energy intake (r = 0.446, *p* = 0.020) in women. For both women and men, the HEI was negatively corrected with BMI (women: r = −0.391, *p* = 0.044; men: r = −0.406, *p* = 0.001). MEDAS and HEI showed a positive correlation with each other (r = 0.352, *p* = 0.002) in men. A positive correlation was found in men for MEDAS with self-control (r = 0.293, *p* = 0.011) and ecological (r = 0.479, *p* = 0.001) and a negative correlation for conservative party preferences (r = −0.230, *p* = 0.047). Meanwhile, HEI showed a positive correlation with age (r = 0.301, *p* = 0.009) and a negative correlation with club membership (r = −0.228, *p* = 0.049) and time discounting (r = −0.250, *p* = 0.030) Table 2.

### 3.4. Association of Social Behaviour and Nutrition Patterns and the Effect of Moderation by Sex

**Figure 2:**
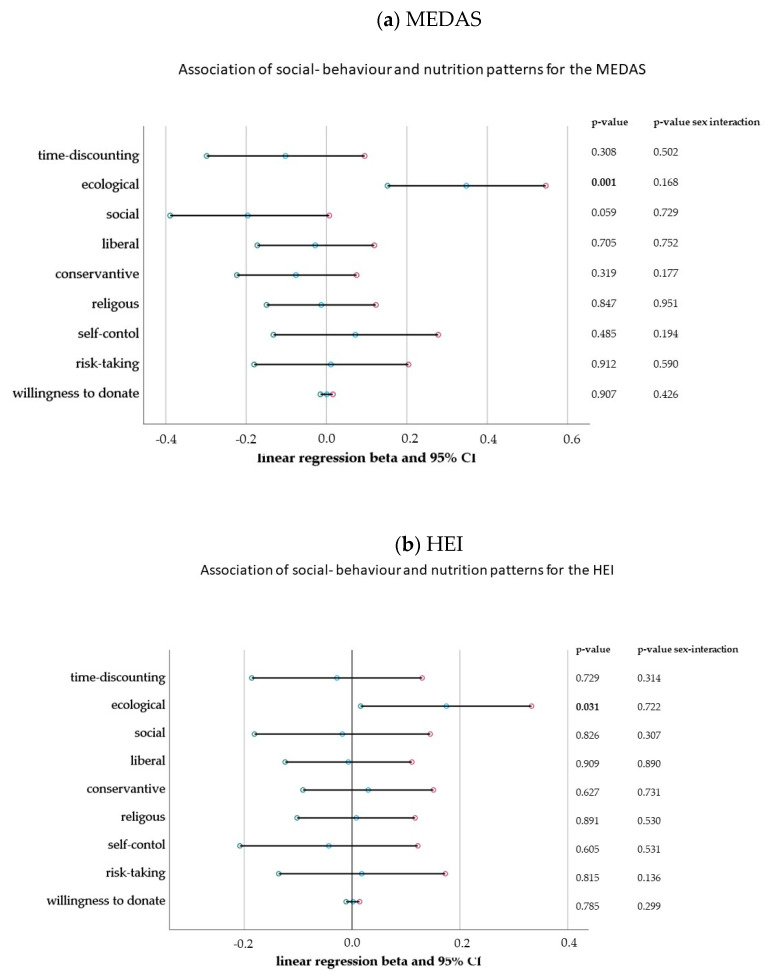
(**a**) Association of social behaviour items with the MEDAS. Linear regression model: R2 = 0.200; *p* = 0.011. Detailed numerical results are presented in Supplementary Tables S6 and S8. (**b**) Association of social behaviour items with the HEI. Significant associations (*p* < 0.05) marked in bold. Linear regression model: R2 = 0.082; *p* = 0.515. Detailed numerical results are presented in Supplementary Tables S5 and S7. Significant associations (*p* < 0.05) marked in bold.

For MEDAS, the social behaviour model was statistically significant; it explained 20% of the variation in the score. The results of the linear regression in Figure 2a showed that the preference for ecological behaviour (beta = 0.348, 95% CI 0.151–0.546, *p* = 0.001) was significantly associated with higher scoring in the MEDAS. A preference for an ecological party explained 13.9% of the model variation. A statistically borderline negative association was shown for the preference of social politics preferences (Beta = −0.196, 95% CI −0.399–0.007, *p* = 0.059).

Similarly, ecological politics preferences were significantly associated with the HEI (beta = 0.175, 95% CI 0.02–0.33, *p* = 0.031). However, the overall social behaviour model was not significant and only explained 8.2% of the variation in HEI (F (9.92) = 0.917, *p* = 0.515) Figure 2b. We did not observe any sex interactions for social behaviour items. Detailed results are shown in Supplementary Tables S5 and S6.

## 4. Discussion

In our cross-sectional study based on a contemporary cohort of individuals with a low cardiovascular risk factor burden, we investigated the associations between dietary patterns and social behaviour including personality traits (self-control, risk taking), political preferences (conservative, liberal, ecological, social) and altruism (willingness to donate, club membership, time discounting) in men and women.

### 4.1. Main Findings and Implications for Future Investigations

Overall, we could demonstrate some sex-specific differences in dietary patterns. Further, we showed that social behaviour such as personality traits, political preferences and altruism explain between 8.2 and 20% of the variability in dietary patterns. In the association analyses, an ecological party preference was consistently related to MEDAS and HEI. The findings provide behavioural insights into the field of (un)healthy food choices that can support the development of behavioural primary prevention strategies.

### 4.2. Dietary Patterns and Sex

Our findings support the assumption that dietary patterns differ between women and men, and we showed that different social behaviour patterns and personal traits are associated with dietary patterns for both sexes. The results underpin the importance of sex differences in dietary patterns as a modifiable risk factor for possible disease development [25]. Recent studies reported more detailed results concerning sex differences for adherence to dietary recommendations using a large sample size of 210,106 women and men [26] and for food preferences and their implications for promoting sustainable dietary patterns in a systematic review [27]. Our study provided detailed information of sex-specific dietary patterns by using two well-established scores, MEDAS and HEI.

Both indices, MEDAS and HEI, are comprehensive assessments of diet quality for this study population. In particular, we observed a lower HEI score for participants with a higher BMI for both sexes, and for MEDAS, we showed that a higher BMI also decreased the scoring in men, but not in women. Although HEI is primarily a measure of overall diet quality, it may also be a predictor of obesity. Other studies have also demonstrated that dietary consumption that follows the HEI is associated with a lower risk for obesity [28]. Sex differences exist in the regulation of energy homeostasis, with a greater intake of energy in men [26,29]. We estimated that these factors were less revenant for the differences in energy intake between women and men given that almost all female participants were in menopause. However, a relationship with higher energy intake in men compared to women has been identified in this study. Factors such as the higher energy needs of men due to a larger body surface, more muscle mass and usually more sports compared to women might have a influence on this result. Despite other study results that showed that lower energy intake is associated with healthy eating behaviours [30], in our cohort, we observed that women with a higher energy intake had higher HEI scores. It is possible that this observation could be explained by the low sample size of women within our study. Consistent with previous studies, men reported higher consumption of alcohol compared to women [31]. In the latest global status report on alcohol and health of the WHO in 2018, 54% men (1.46 billion) and 32% of women (0.88 billion) aged 15 and older worldwide consumed alcohol. Men experience an estimated 2.3 million deaths and 106.5 million DALYs attributable to alcohol consumption, while women experience 0.7 million deaths and 26.1 million DALYs attributable to alcohol consumption [32]. Thus, the assessment of alcohol as an important indicator for dietary patterns shows potential to detect and prevent unhealthy eating habits in consideration of sex-specific patterns of consumption in low-risk individuals. According to average scores achieved for items in the MEDAS and items in the HEI, women scored significantly lower for fruits and wine, but significantly higher for vegetables, compared to men. The low fruit consumption in women is in contrast to other studies, where women compared to men had a higher intake of fruits [33,34]. However, the tendency of healthy dietary patterns such as less or no alcohol consumption and higher intake of vegetables in women within this cohort is mainly in line with the results of other established population studies [26,33]. Thus, the low consumption of fruit could possibly be explained by, again, the low sample size of women and the generally lower overall food consumption in women rather than in men.

In our study, women had a higher burden of dyslipidaemia compared to men and also scored significantly higher for butter, margarine and cream consumption, which is known to increase total and LDL cholesterol [35]. As opposed to our findings, few studies show that women tend to have more favourable levels of blood cholesterol compared to men [36,37]. The onset of dyslipidaemia occurs later in women, and often is more poorly controlled compared to men [38].

### 4.3. Social Behaviour, Personality Traits and Political Preferences

In general, we showed that men were frequently more active members in a club, self-control correlated with an increase in the MEDAS scoring, and we identified significance in the preference for conservative politics as compared to women.

We showed that, in men, active club membership correlated with a higher MEDAS and HEI score. An active role in the community is a strong indicator of social support and amplifies integration into society. In a few studies, social factors such as informal networks have been identified to have an influence on food-related behaviours [39,40,41]. If we assume that active participation in a club helps to avoid social isolation, our findings are in line with previous studies.

Self-control, as a major personality characteristic, explains several health-related behaviours [42]. We did not observe an association between self-control and dietary patterns in the regression analysis. However, self-control was correlated with MEDAS in men. Other studies support the importance of self-control for healthy eating attitudes and its role in the maintenance of weight/shape concerns and disordered eating for both women and men [43,44].

We identified a significant association between the preference for ecological-oriented politics and the tendency towards healthy dietary patterns for both indices. Ecological behaviour is a pro-environmental attitude and it refers to the human relationship with the natural environment and is a complex, diverse and dynamic phenomenon, and it seems that ecological behaviour is a significant factor in lifestyle management and food choices. The literature suggests that environment and exposure can predict food-related health risk behaviours and health outcomes [45]. Altruism, tested by the willingness to donate, showed no sex-specific differences, despite a significant difference in income between men and women. There have been a few investigations with the objective to identify associations of altruistic behaviour for recruitment and enrolment optimisations in RCTs [46,47]. Weissberger and colleagues identified, for example, that increased financial altruism is associated with disease occurrence in older adults [48]. Conversely, Shim and colleagues incorporated altruism into a game-theoretic epidemiological model to determine how altruistic behaviour impacts the disease burden. They recommended promoting altruism to improve public health outcomes [49]. In the association analysis, we could not find an interaction effect of sex between dietary patterns and social behaviour items. However, it should be considered that a number of gender-based stereotypes about food exist in every human culture. Although the causes of this are far from being fully elucidated, the consequences for food choices and dietary habits might be relevant because both men and women tend to adhere to those expectations most likely to reinforce their own gender identities [50,51].

### 4.4. Recommendations for Primary Prevention

■ The association of ecological preferences and healthy dietary patterns inform preventive agendas to focus on different behavioural strategies to promote environmentally sustainable food consumption in high-income countries.
■ Health aspects are not the only determinants of food choices. People may have various food-related goals, such as to save money or maintain a sustainable lifestyle, which are often more salient and compete with the importance of health considerations.
■ Social characteristics such as self-control and ecological preferences could support the ability to reflect on the influence of environmental factors such as marketing and peers. To educate individuals about this, behavioural insights that could support healthy dietary patterns could be integrated into behavioural primary prevention strategies.

The importance of the determination of human health behaviours, investigation of personality traits and preferences and consequently the development of appropriate prevention programs has been underlined by prior research [52].

### 4.5. Strengths and Limitations

Our results are limited by the cohort approach, which does not permit statements on causality. Bias may exist in the form of recall bias for food items and social desirability bias. The relationship between dietary patterns and social behaviour is complex and we identified weak associations. Therefore, we accounted for possible sex differences and incomes, but may have missed other relevant factors, such as education levels. We had to deal with a relatively small sample size (especially for women). Despite careful adjustment, residual confounding may exist. Additionally, the present research was restricted to the metropolitan region of Hamburg in Germany, and the results may not be generalisable to other populations. The main strength of our study is a well-characterised and consciously selected cohort with low-risk participants, where we were able to identify at least weak differences for social behaviours in men and women. The other strength is the advantageous comprehensive assessment of dietary patterns with different items based on protocols from both the Mediterranean Diet Adherence Screener and the validated Healthy Eating Index and the provision of a broader picture of food consumption.

## 5. Conclusions

In our cohort of low-risk individuals, we could demonstrate that social behaviour such as personality traits (self-control, risk taking), political preferences (conservative, liberal, ecological, social) and altruism (willingness to donate, club membership, time discounting) may be related to dietary patterns. In particular, ecological preferences showed a significant association with healthy dietary habits.

However, the observed associations were weak. There were no sex differences observed between social behaviour and dietary patterns. Based on our analyses, primary prevention might address behavioural aspects in order to improve dietary habits and thus health in both women and men.

## Figures and Tables

**Table 1:** Characteristics of study participants.

Variables	Total (N = 102)	Women (*n* = 27)	Men (*n* = 75)	Standardised Test Statistic	*p*-Value
**Sociodemographic and clinical data**					
Age, years	62.4 (53.6, 69.1)	62.4 (56.6, 70.3)	63.5 (53.3, 69.0)	−0.0618 ^c^	0.536
Income categories					
Low (<EUR 2500)	16 (15.5%)	**7 (25.9%)**	**9 (12.0%)**	**10.268 ^b^**	**0.008**
Middle (EUR 2500 < EUR 5000)	56 (54.9%)	**18 (66.7%)**	**38** **(50.7%)**		
High (>EUR 5000)	30 (29.4%)	**2 (7.4%)**	**28 (37.3%)**		
Body mass index, kg/m^2^	25.5 (23.1, 27.8)	25.0 (23.1, 27.3)	25.4 (23.1, 27.8)	0.155 ^c^	0.876
Body mass index categories					
Underweight	1 (1.0%)	-	1 (1.4%)	0.985 ^b^	0.875
Normal weight	48 (47.1%)	14 (51.9%)	33 (44.6%)		
Overweight	46 (45.1%)	11 (40.7%)	36 (47.3%)		
Obesity	7 (7.9%)	2 (7.4%)	5 (6.8%)		
**Prevalent diseases**					
Type 2 diabetes mellitus	2 (2.0%)	-	2 (2.7%)	1.421 ^a^	0.153
Arterial hypertension	22 (21.6%)	8 (29.6%)	14 (18.7%)	1.410 ^a^	0.235
atrial fibrillation	52 (51%)	14 (51.0%)	38 (50.7%)	0.011 ^a^	0.916
Dyslipidaemia	21 (20.6%)	**10 (37.0%)**	**11 (14.7%)**	**6.077 ^a^**	**0.014**
**Lifestyle factors**					
MEDAS, points	3 (1, 4)	3 (1, 5)	2 (2, 4)	−0.320 ^c^	0.749
Healthy Eating Index, points	54.9 (47.3, 60.3)	55.6 (48.6, 61,8)	54.8 (67.2, 59.3)	−0.804 ^c^	0.421
Healthy Eating Index, categories					
Poor (≤40 pts.)	10 (9.8%)	1 (13.7%)	9 (12.0%)	1.355	0.556
Improvable (>40–64 pts.)	77 (75.5%)	22 (80.4%)	55 (73.3%)		
Good (>64 pts.)	15 (14.7)	4 (5.9%)	11 (14.7%)		
Diet change past 12 months					
No	82 (80.4%)	22 (78.4%)	60 (80.0%)	0.028 ^a^	0.868
Yes, partially	20 (19.6%)	5 (18.5%)	15 (20.0%)		
Energy intake, kcal/day	2187 (1904, 2504)	**2138 (1224, 2418)**	**2253 (1974, 2583)**	**3.243**	**0.001**
Physical activity, MET-h/day (in Log10)	3.3 (3.1, 3.6)	3.5 (3.1, 3.8)	3.3 (3.1, 3.5)	1.737 ^c^	0.082
Alcohol consumption	82 (80.4)	**18 (66.7%)**	**63 (84.0%)**	**4.249 ^a^**	**0.039**
Smoking					
Current	62 (60.8%)	6 (22.2%)	10 (13.3%)	2.018 ^b^	0.379
Former		9 (33.3%)	41 (54.7%)		
**Social behaviour**					
*Personality traits*					
Risk taking	6 (5.7)	6 (5.6)	6 (5.8)	10.563 ^b^	0.250
Self-control	9 (8.10)	8 (7.8)	9 (8.10)	12.425 ^b^	0.139
Religiousness	4 (1.6)	4 (1.5)	4 (1.7)	10.468 ^b^	0.276
*Political preferences*					
Conservative	5 (2.7)	**4 (1.5)**	**6 (4.8)**	**17.628 ^b^**	**0.032**
Liberal	6 (2.7)	5 (2.6)	6 (4.8)	14.993 ^b^	0.088
Social	8 (6.9)	8 (7.9)	8 (6.9)	6.444 ^b^	0.826
Ecological	8 (7.9)	8 (7.9)	8 (6.9)	6.670 ^b^	0.783
*Altruism*					
Willingness to donate in EUR (EUR 0 to 50)	EUR 20 ± 22	25 (20.50)	20 (20.50)	−0.580 ^c^	0.562
Low willingness to donate (EUR 0–25)	61 (59.8%)	16 (59.3%)	45 (60.0%)	0.005 ^a^	0.946
High willingness to donation (EUR 25–50)	41 (41.2%)	11 (40.7%)	30 (40.0%)		
Club membership					
No	37 (36.3%)	17 (63.3%)	20 (26.7%)	10.655 ^b^	**0.004**
Yes, passive	12 (11.8%)	2 (7.4%)	10 (13.3%)		
Yes, active	53 (52.0%)	8 (29.6)	45 (60.0%)		
Time discounting	4 (2.6)	3 (2.4)	4 (3.5)	3.175 ^b^	0.957

Note. Data presented as percentages for categorical variables and as median (1st quartile, 3rd quartile) for continuous variables. ^a^ Pearson’s Chi-square test. ^b^ Fisher’s exact test. ^c^ Mann–Whitney U test. Multiple data imputation with five imputations was performed to fill in missing values of income (*n* = 14), and based on N = 102. Significant correlations (*p* < 0.05) marked in bold.

**Table 2:** Correlations of MEDAS and HEI with independent variables by sex.

Independent Variables	MEDAS				HEI			
	Women		Men		Women		Men	
	R	*p*-Value	R	*p*-Value	R	*p*-Value	R	*p*-Value
**Sociodemographic and clinical data**								
Age, years	−0.042	0.836	0.104	0.375	0.037	0.854	**0.301**	**0.009**
Income	0.078	0.698	−0.009	0.942	0.014	0.889	−0.069	0.555
Body mass index, kg/m^2^	−0.077	0.702	**−0.432**	**0.001**	**−0.391**	**0.044**	**−0.406**	**0.001**
**Lifestyle factors**								
MEDAS, points	-	-	-	-	0.307	0.119	**0.352**	**0.002**
Healthy Eating Index, points	0.307	0.119	**−0.352**	**0.002**	**-**	**-**	-	-
Diet change past year	0.257	0.196	0.184	0.114	−0.037	0.856	0.050	0.670
Energy intake, kcal/day	0.003	0.986	−0.010	0.932	**0.446**	**0.020**	0.165	0.158
Physical activity, MET-h/day	0.233	0.242	0.089	0.448	−0.255	0.199	−0.065	0.577
**Social behaviour**								
*Altruism*								
Willingness to donate	0.112	0.577	−0.032	0.783	0.252	0.206	−0.007	0.955
Club membership	0.359	0.066	0.038	0.745	0.192	0.337	**−0.228**	**0.049**
Time discounting	−0.290	0.142	−0.218	0.060	0.191	0.341	**−0.250**	**0.030**
*Personality traits*								
Risk taking	−0.221	0.268	0.103	0.379	0.188	0.348	0.014	0.905
Self-control	0.026	0.898	**0.293**	**0.011**	0.093	0.646	0.153	0.326
Religiousness	−0.025	0.900	0.029	0.804	0.140	0.487	0.075	0.521
*Political preferences*								
Conservative	0.026	0.899	**−0.230**	**0.047**	0.146	0.486	0.45	0.700
Liberal	0.035	0.862	0.029	0.802	0.074	0.714	0.145	0.214
Social	0.110	0.584	0.165	0.158	0.173	0.387	0.097	0.407
Ecological	0.155	0.441	**0.479**	**0.001**	0.048	0.812	0.244	0.053

Spearman correlation coefficients of MEDAS and HEI with independent variables (*n* = 102). BMI, body mass index; MET, metabolic equivalents. Significant correlations (*p* < 0.05) marked in bold.

## Data Availability

The data analysed during the current study are not publicly available due to the German National Data Protection Regulation. They are available on reasonable request from the corresponding author.

## Supplementary Materials

The following supporting information can be downloaded at: https://www.mdpi.com/article/10.3390/nu15081832/s1, Table S1: Assessment of social behaviour, Table S2: Sex differences in food items of the Mediterranean Diet Adherence Screener, Table S3: Sex differences in the food items of the Healthy Eating Index (HEI), Table S4: Differences individuals with low and high willingness to donate in the food items of the Mediterranean Diet Adherence Screener, Table S5: Linear regressions of social behaviour variables with HEI, Table S6: Linear regressions of social behaviour variables with MEDAS, Table S7 Moderation effect of sex for social behaviour with HEI, Table S8: Moderation effect of sex for social behaviour with MEDAS, Figure S1: Dietary patterns of individuals with high (N = 61) and low (N = 41) willingness to donate according to average scores achieved for items in the Mediterranean Diet Adherence Screener (MEDAS).
