# Supplementary material for: Sex-Specific Dietary Patterns and Social Behaviour in Low-Risk Individuals
Source: Nutrients. 2023 Apr 11;15(8):1832. doi: 10.3390/nu15081832 (PMC10146290; doi:10.3390/nu15081832)
Supplement: Supplementary file 1 [file nutrients-15-01832-s001.zip › nutrients-2307436-supplementary.pdf]

Manuscript Supplement

Supplementary Table S1 Assessment of social behaviour

**Assessment instruments**	**Study variable**
Questionnaire (self-administered)	Gender, age, income, dietary changes within a year prior to examination, alcohol consumption, current and former smoking status
Questionnaire (self-administered) and patient's file	Prevalent diseases (type 2 diabetes mellitus, arterial hypertension, dyslipidaemia)
Patient's file	Height, medication (blood-thinning medications: Phenprocuomon, Warfarin, and further; anticoagulants: Dabigantran, Apixaban, Rivaroxaban, and further)
Calibrated mechanical column scale (Seca 709)	Weight
Blood sampling	Blood samples were taken and NT-proBNP (pg/mL) was determined as a cardiovascular risk related blood marker (Elecsys® proBNP II, Roche).
12-channel electrocardiogram (Schiller® CARDIOVIT AT-10 plus)	Resting-heart rate
Questionnaire (IPAQ-SF)	Physical activity
Three-day dietary record	Dietary intake (energy intake, Mediterranean Diet Adherence Screener (MEDAS), Healthy Eating Index (HEI)
**Social behaviour Items (scale from 0 (none) to 10 (strongly agree))**	
**Personality traits**	
risk taking	Are you generally a risk-taker, or do you try to avoid risk?
self-control	When I set my mind to something, I follow through.
religiousness	How strongly religious do you consider yourself to be?
**Political preferences**	
conservative	I identify myself with conservative-oriented politics
liberal	I identify myself with liberal-oriented politics
social	I identify myself with social-oriented politics
ecological	I identify myself with ecological-oriented politics
**Altruism**	
donation willingness	From my 50 Euro expense allowance I would like to donate __ Euro to UNICEF.
club membership	Are you an active and/or passive member in a club
time-discounting	In general, are you willing to give up something today in order to benefit from something in the future?

Supplementary Table S2 Sex differences in food items of the Mediterranean Diet Adherence Screener

**MEDAS Items**	**Women (N=27)**	**Men (N=75)**	**Standardised Test Statistic**	**p-value**
Olive Oil/Rapeseed Oil as main fat source for cooking	4 (14.8)	3 (4.0)	3.633	0.057
Vegetables	5 (18.5)	11 (14.8)	0.223	0.637
Fruit	**1 (3.7)**	**15 (20.0)**	**3.986**	**0.046**
Red meat, hamburger, sausages	13 (48.9)	25 (33.3)	1.864	0.172
Butter, margarine, cream	**11 (40.7)**	**9 (12.0)**	**10.301**	**0.001**
Sugar-sweetened beverages	16 (59.3)	46 (61.3)	0.036	0.850
Wine	**3 (11.1)**	**24 (32.0)**	**4.451**	**0.035**
Pulses	3 (11.1)	4 (5.3)	1.037	0.309
Fish, seafood	3 (11.1)	17 (22.7)	1.682	0.195
Pastry, such as cookie or cake	3 (11.1)	14 (18.7)	0.816	0.366
Nuts	8 (29.6)	14 (18.7)	1.410	0.235
Prefer white meat over red meat	6 (22.2)	18 (24.0)	0.035	0.852
Dishes with a sauce of tomato, garlic, onion sautéed in olive oil	3 (11.1)	14 (13.7)	0.210	0.645
*MEDAS Score	3 (1,5)	3 (2,4)	0.320	0.749
Note. Data presented as amount (percent) and test statistics of Pearson's Chi Square. *Data presented as median (1st quartile, 3 rd quartile). Mann-Whitney U tests.				

Supplementary Table S3 Sex differences in the food items of the Healthy Eating Index (HEI)

**HEI Items**	**Women (N=27)**	**Men (N=75)**	**Standardised Test Statistic**	**p-value**
Beverages	8.8 (5.7, 11.8)	8.5 (5.7, 11.8)	1.066	0.287
Vegetables	**5.1 (3.6, 7.1)**	**3.4 (2.0, 5.6)**	**2.811**	**0.005**
Fruit	8.0 (4.8, 12.1)	9.1 (4.5, 12.8)	0.307	0.759
Cereal and potato	6.8 (4.9, 8.1)	7.5 (6.2, 8.5)	1.597	0.110
Dairy	6.0 (4.6, 8.9)	6.1 (4.2, 7.9)	0.979	0.328
Meat, sausage, fish, egg	5.0 (3.7, 7.2)	4.3 (2.9, 5.8)	1.442	0.149
Fat, oil	7.2 (5.2, 8.9)	6.1 (5.0, 7.9)	1.043	0.149
Sweets, foods high in fat	6.1 (4.4, 9.6)	7.6 (5.3,10.0)	1.285	0.199
Score	55.6 (48.6, 61.8)	54.8 (47.2, 59.3)	0.804	0.421
Note. Data presented as median (1st quartile, 3 rd quartile). Mann-Whitney U tests.				

Supplementary Table S4 Differences individuals with low and high willingness to donate in the food items of the Mediterranean Diet Adherence Screener

**Variables**	**Total (N=102)**	**Low Donors (N= 61)**	**High Donors (N=41)**	**p-value**
Olive Oil/Rapeseed Oil as main fat source for cooking	7 (6.9)	4 (6.6)	3 (7.3)	0.882
Vegetables	16 (15.7)	9 (14.8)	7 (17.1)	0.752
Fruit	16 (15.7)	12 (19.7)	4 (9.8)	0.177
Red meat, hamburger, sausages	38 (37.3)	20 (32.8)	18 (43.9)	0.255
Butter, margarine, cream	20 (19.6)	13 (21.3)	7 (17.1)	0.597
Sugar-sweetened beverages	62 (60.8)	40 (65.6)	22 (53.7)	0.227
Wine	27 (26.5)	19 (31.1)	8 (19.5)	0.192
Pulses	7 (6.9)	4 (6.6)	3 (7.3)	0.882
Fish, seafood	20 (19.6)	14 (23.0)	6 (14.6)	0.300
Pastry, such as cookie or cake	17 (16.7)	7 (11.5)	10 (24.4)	0.086
Nuts	22 (21.6)	16 (26.2)	6 (14.6)	0.163
Prefer white meat over red meat	24 (23.5)	**10 (16.4)**	**14 (34.1)**	**0.034**
Dishes with a sauce of tomato, garlic, onion sautéed in olive oil	14 (13.7)	**11 (18.0)**	**3 (7.3)**	**0.056**

Note. Data presented as number (percent) and test statistics of Pearson's Chi Square. [a] No statistics could be computed for Olive Oil/Rapeseed Oil

Supplementary Table S5 Linear regressions of social behaviour variables with HEI

Model		Unstandardized Coefficients		Standardized Coefficients			95,0% Confidence Interval for B		Correlations			Collinearity Statistics	
		B	Std. Error	Beta	t	Sig.	Lower Bound	Upper Bound	Zero-order	Partial	Part	Tolerance	VIF
	donation	.002	.006	.029	.274	.785	-.011	.014	.051	.029	.027	.888	1.126
	risk-taking	.018	.078	.027	.235	.815	-.136	.173	.037	.024	.023	.756	1.322
	self-control	-.043	.083	-.060	-.519	.605	-.208	.122	.051	-.054	-.052	.748	1.337
	religiousness	.008	.055	.016	.138	.891	-.102	.117	.076	.014	.014	.756	1.322
	conservative	.030	.061	.063	.488	.627	-.091	.151	.001	.051	.049	.600	1.668
	liberal	-.007	.059	-.014	-.115	.909	-.124	.111	.060	-.012	-.011	.692	1.445
	social	-.018	.082	-.030	-.221	.826	-.181	.145	.146	-.023	-.022	.547	1.828
	ecological	.175	.080	.305	2.190	.031	.016	.333	.271	.223	.219	.514	1.946
	time-discounting	-.028	.080	-.040	-.347	.729	-.186	.130	-.103	-.036	-.035	.747	1.339

a. Dependent Variable: HEI score modified

Supplementary Table S6 Linear regression of social behaviour variables with MEDAS

Model	Unstandardized Coefficients		Standardized Coefficients			95,0% Confidence Interval for B		Correlations			Collinearity Statistics	
	B	Std. Error	Beta	t	Sig.	Lower Bound	Upper Bound	Zero-order	Partial	Part	Tolerance	VIF
donation	.001	.008	.012	.118	.907	-.015	.016	-.021	.012	.011	.888	1.126
risk-taking	.011	.097	.012	.110	.912	-.182	.204	.050	.012	.010	.756	1.322
self-control	.072	.103	.076	.700	.485	-.133	.278	.169	.073	.065	.748	1.337
religiousness	-.013	.069	-.021	-.193	.847	-.150	.123	.028	-.020	-.018	.756	1.322
conservative	-.076	.076	-.121	-1.001	.319	-.227	.075	-.161	-.104	-.093	.600	1.668
liberal	-.028	.074	-.043	-.380	.705	-.175	.119	.007	-.040	-.035	.692	1.445
social	-.196	.102	-.241	-1.914	.059	-.399	.007	.105	-.196	-.178	.547	1.828
ecological	.348	.099	.456	3.504	.001	.151	.546	.373	.343	.327	.514	1.946
time-discounting	-.102	.099	-.111	-1.025	.308	-.299	.095	-.213	-.106	-.096	.747	1.339

a. Dependent Variable: MEDAS score

Supplementary Table S7 Moderation effect of sex for social behaviour with HEI

	interaction - p-value	R^2	OR per SD (95% CI)	p-value
willingness to donate	0.299	0.021	-0.014 (-0.410/0.130)	0.560
risk-taking	0.136	0.032	-0.272 (-0.632/0.870)	0.359
self-control	0.531	0.016	0.088 (-0.240/0.416)	0.660
religiousness	0.530	0.019	-0.076 (-0.317/0.165)	0.604
conservative	0.731	0.010	-0.043 (-0.290/0.204)	0.804
liberal	0.890	0.014	-0.016 (-0.246/0.214)	0.707
social	0.307	0.036	-0.126 (-0.401/0.148)	0.307
ecological	0.722	0.078	0.044 (-0.202/0.291)	**0.046**
time-discounting	0.314	0.027	-0.171 (-0.508/0.165)	0.438

Supplementary Table S8 Moderation effect of sex for social behaviour with MEDAS

	interaction - p-value	R^2	OR per SD (95% CI)	p-value
willingness to donate	0.426	0.008	-0.015 (-0.510/0.022)	0.856
risk-taking	0.590	0.007	0.132 (-0.354/0.619)	0.882
self-control	0.194	0.051	0.284 (-0.146/0.714)	0.162
religiousness	0.951	0.002	0.010 (-0.335/0.314)	0.981
conservative	0.177	0.035	-0.149 (-0.475/0.177)	0.322
liberal	0.752	0.002	-0.049(-0.358/0.259)	0.977
social	0.729	0.013	-0.065 (-0.436/0.306)	0.736
ecological	0.168	0.156	0.221 (-0.095/0.536)	**0.001**
time-discounting	0.502	0.050	0.151 (-0.293/0.595)	0.168

Supplementary Figures

Supplementary Figure S1.

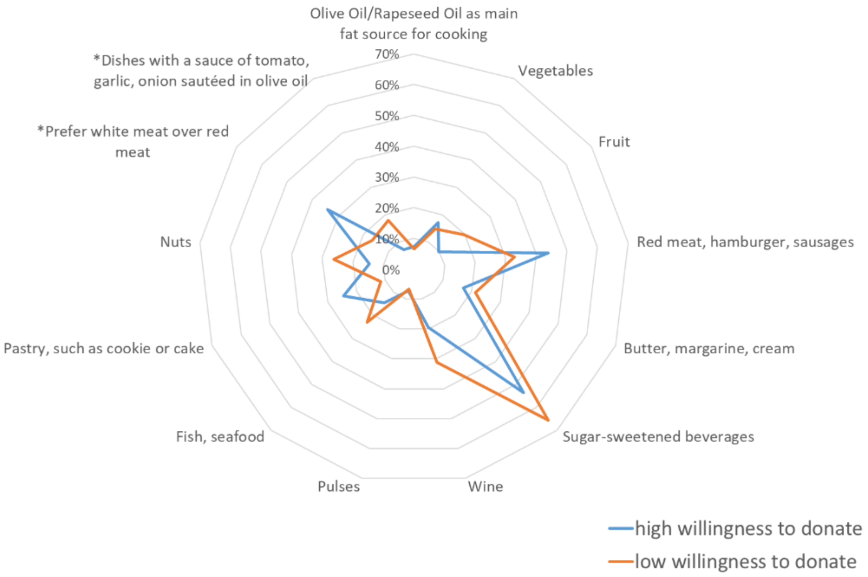

Dietary patterns of individuals with high (N=61) and low (N=41) willingness to donate according to average scores achieved for items in the Mediterranean Diet Adherence Screener (MEDAS). Note. The category "Olive oil, rapeseed oil" was excluded as no participant scored. Scores ranged. Scores ranged presented in percentages. * $p < 0.05$.
